# A new era in chronic spontaneous urticaria: FDA approval of the oral BTK inhibitor remibrutinib

**DOI:** 10.1097/MS9.0000000000004298

**Published:** 2025-11-24

**Authors:** Mahnoor Jan, Khola Qazi, Aliya Noor, Mawra Naveed, Maliha Khalid, Aminath Waafira

**Affiliations:** a Department of Medicine, Shaikh Khalifa Bin Zayed Al Nahyan Medical and Dental College, Lahore, Pakistan; b Department of Medicine, Jinnah Sindh Medical University, Karachi, Pakistan; c Department of Medicine, King Edward Medical University, Lahore, Pakistan; d Department of Medicine, The Maldives National University, Malé, Maldives

**Keywords:** BTK inhibitor, chronic spontaneous urticaria, mast cells, remibrutinib, targeted therapy

## Abstract

On 30 September 2025, the U.S. Food and Drug Administration (FDA) approved Rhapsido® (remibrutinib) as the first targeted oral therapy for chronic spontaneous urticaria (CSU) in adults who remain symptomatic despite H1-antihistamine treatment. This approval marks a major advancement in CSU management, offering a convenient, precision-based alternative to injectable biologics such as omalizumab. CSU is a chronic, relapsing inflammatory skin disorder characterized by recurrent wheals and angioedema, often accompanied by sleep disturbance, emotional distress, and reduced quality of life. The pathophysiology involves abnormal activation of mast cells and basophils through the IgE receptor (FcεRI) and downstream Bruton’s tyrosine kinase (BTK) signaling, leading to histamine and cytokine release. Remibrutinib, a potent and selective covalent BTK inhibitor, interrupts this inflammatory cascade at its source, thereby providing upstream control of disease activity. FDA approval was based on Phase III REMIX-1 and REMIX-2 trials, which demonstrated rapid, significant, and sustained improvements in Urticaria Activity Score (UAS7), Itch Severity Score (ISS7), and Hives Severity Score (HSS7) compared with placebo. Approximately one-third of patients achieved complete symptom resolution by week 12, and the drug showed an acceptable safety profile with mostly mild adverse events and no need for routine laboratory monitoring. The introduction of remibrutinib represents a paradigm shift in CSU therapy, transitioning from symptomatic relief toward immune-targeted, patient-friendly oral treatment. Future studies should evaluate long-term safety, real-world efficacy, and cost-effectiveness to fully define its role in personalized CSU management.

*Dear Editor,*

This letter to the editor adheres to the TITAN guideline on the need for transparency in AI use in healthcare^[1]^. On 30 September 2025, the U.S. Food and Drug Administration (FDA) approved Rhapsido® (remibrutinib) as an oral therapy for chronic spontaneous urticaria (CSU) in adult patients who remain symptomatic despite treatment with H1-antihistamines. This approval represents the first targeted oral therapy with a novel mechanism of action for CSU, marking a paradigm shift in treatment by moving beyond symptom control toward upstream immune pathway modulation^[2]^.

CSU is a distressing dermatologic disorder characterized by recurrent pruritic wheals and angioedema persisting for 6 or more weeks without an identifiable cause. It affects 0.2 to 0.3% of the U.S. population and causes impaired sleep, emotional distress, and reduced quality of life^[3]^. CSU results from the abnormal activation of skin mast cells and basophils via the high-affinity IgE receptor (FcεRI) and downstream signaling, including Bruton’s tyrosine kinase (BTK), leading to the release of histamine and other pro-inflammatory mediators. The first-line treatment of CSU traditionally includes second-generation H1-antihistamines, often at increased doses, followed by omalizumab, an anti-IgE monoclonal antibody, as a second-line in non-responders; however, more than half of patients remain symptomatic, and injectable therapies pose adherence and cost challenges^[4]^. These limitations highlight the pressing need for an effective, safe, and easily administered oral therapy.

Remibrutinib is a highly selective, oral Bruton’s tyrosine kinase (BTK) inhibitor that targets mast cells and basophils, the main effector cells driving inflammation in CSU. It has the potential to block the inflammatory pathway that leads to the release of histamine and cytokines by covalently inhibiting BTK, thereby providing symptom relief. As the first and only oral, targeted BTKi approved for CSU, it offers a convenient and innovative alternative to injectable biologics such as omalizumab, representing a major step toward precision-driven, immune-targeted therapy^[5]^.

The FDA approval of remibrutinib was based on the results from pivotal Phase III trials REMIX-1 and REMIX-2, both of which met all primary endpoints^[2]^. Patients treated with remibrutinib demonstrated a statistically significant and clinically meaningful improvement in weekly Urticaria Activity Score (UAS7), Itch Severity Score (ISS7), and Hives Severity Score (HSS7) in comparison to those on placebo. Symptom relief began as early as Week 2, and approximately one-third of patients had complete relief from itch and hives (UAS7 = 0) by Week 12^[6]^.

Remibrutinib was well tolerated overall, with the most frequently reported adverse effects being nasopharyngitis, headache, nausea, and abdominal pain. A slightly higher rate of upper respiratory tract infections was also noted. Importantly, no routine laboratory monitoring is required, enhancing its practicality in long-term use^[2,6]^.

The approval of Rhapsido® (remibrutinib) marks an important milestone in CSU care by providing a fast-acting, targeted, and convenient oral therapy that could revolutionize current treatment algorithms and improve patient quality of life. Future research should focus on evaluating long-term safety, sustained efficacy, cost-effectiveness, and real-world outcomes to optimize its role in individualized CSU management.

## Data Availability

No data were generated for this manuscript.
